# The Atacama Rover Astrobiology Drilling Studies (ARADS) Project

**DOI:** 10.1089/ast.2022.0126

**Published:** 2023-12-20

**Authors:** B. Glass, D. Bergman, V. Parro, L. Kobayashi, C. Stoker, R. Quinn, A. Davila, P. Willis, W. Brinckerhoff, K. Warren-Rhodes, M.B. Wilhelm, L. Caceres, J. DiRuggiero, K. Zacny, M. Moreno-Paz, A. Dave, S. Seitz, A. Grubisic, M. Castillo, R. Bonaccorsi

**Affiliations:** ^1^ NASA Ames Research Center, Moffett Field, California, USA.; ^2^ Honeybee Robotics, Pasadena, California, USA.; ^3^ Centro de Astrobiología (CAB), CSIC-INTA, Torrejon de Ardoz, Spain.; ^4^ NASA Jet Propulsion Laboratory, Pasadena, California, USA.; ^5^ NASA Goddard Space Flight Center, Greenbelt, Maryland, USA.; ^6^ SETI Institute, Carl Sagan Center, Mountain View, California, USA.; ^7^ University of Antofagasta, Antofagasta, Chile.; ^8^ Johns Hopkins University, Baltimore, Maryland, USA.

**Keywords:** Astrobiology, Automated drilling, Biosignatures, Atacama Desert, Analog mission, Search for life

## Abstract

With advances in commercial space launch capabilities and reduced costs to orbit, humans may arrive on Mars within a decade. Both to preserve any signs of past (and extant) martian life and to protect the health of human crews (and Earth's biosphere), it will be necessary to assess the risk of cross-contamination on the surface, in blown dust, and into the near-subsurface (where exploration and resource-harvesting can be reasonably anticipated). Thus, evaluating for the presence of life and biosignatures may become a critical-path Mars exploration precursor in the not-so-far future, circa 2030. This Special Collection of papers from the Atacama Rover Astrobiology Drilling Studies (ARADS) project describes many of the scientific, technological, and operational issues associated with searching for and identifying biosignatures in an extreme hyperarid region in Chile's Atacama Desert, a well-studied terrestrial Mars analog environment. This paper provides an overview of the ARADS project and discusses in context the five other papers in the ARADS Special Collection, as well as prior ARADS project results.

## 1. Introduction

The availability of water is one of the most critical parameters that influence the biological potential of planetary environments—both as a medium for prebiotic chemistry, abiogenesis, and life and as a vehicle for generating geo- and electrochemical systems that can support metabolism (Russell *et al.,* 2014; Clark *et al.,* 2021). Water is also a critical resource for future human exploration and habitation of planetary environments (Sanders *et al.,* 2015; Kleinhenz *et al.,* 2018). Changes in both NASA priorities and announced commercial space intentions toward Mars add more urgency to the search for life and water (Heldmann *et al.,* 2022). Remote sensing and landed assets have established that water ice is widespread near the surface of Mars at mid and high latitudes on both hemispheres (*viz.* Byrne *et al.,* 2009; Dundas *et al.,* 2018; Hibbard *et al.,* 2021; Luzzi *et al.,* 2023). This motivates future missions, such as the proposed Icebreaker mission (McKay *et al.,* 2013) and the recently advocated Mars Life Explorer (NAP, 2022), which intentionally target near-surface ground ice that may contain extant life or signatures of past life.

Seeking evidence of life prior to human arrival is important to preserve signs of any existing or past evidence of martian life to reduce the risk of unintentional forward contamination of martian environments in which terrestrial microbes might be able to gain a foothold (*e.g.,* Hallsworth, 2021 and references therein) and to potentially protect the health of any human crews and enable their safe return to Earth. Human exploration will require a rapid but thorough survey of any local “area of operations” on Mars to determine the spatial distribution, quantity, and quality (*e.g.,* salt/mineral/organic content) of water, as well as evaluate signs of current or extinct life.

Sample acquisition and handling are key elements of robotic missions that target near-surface ground ice. Drilling and sampling devices in those environments must autonomously adjust to uncertain concentrations, depths, and locations of subsurface ice. Drilling and sampling activities on Mars must also contend with a relative lack of *a priori* knowledge of the geophysical conditions in the subsurface, including the presence of hard materials and the physical and chemical properties of samples. For example, the 2007 Phoenix mission experienced sticky and refreezing material in a scoop (Bonitz *et al.,* 2008), which was an impedance to subsequent sample transfer.

There are multiple ways and scenarios in which the geology, hydrology, and astrobiology in a Mars exploration zone (defined in terms of an activity radius around a base or landing zone, as per Bussey and Hoffman [2016]) might be investigated, with potential combinations of rovers, landers, and eventual human crew. The currently dominant robotic exploration paradigm stresses mobility and surveying multiple sites (*e.g.,* rovers with shallow sampling, rather than a lander with a deep drill). The postponed ExoMars mission (ESA, 2022) proposed a rover with a deeper (<2 m) drill, and the upcoming 2024 Lunar Volatiles Investigating Polar Exploration Rover (VIPER) surveying mission (Zacny *et al.,* 2021) will carry a 1 m sampling drill.

In the mid 2000s, the Mars Astrobiology Field Laboratory (AFL) was proposed as a follow-on mission concept to the Mars Science Laboratory Curiosity rover mission (Beegle *et al.,* 2007). The AFL would have included precision drilling to 2 m, automated sample handling, and remote processing of cores. AFL would have incorporated comprehensive planetary protection measures and cross-contamination avoidance, given its goals of potential biosignature or extant life detection. However, at that time automated drilling sampling and processing technology was not considered sufficiently mature, nor were the biosignature-detection instruments or measures for planetary protection and contamination avoidance deemed to be adequately identified or developed. The AFL mission concept was, therefore, sidelined, as attention shifted to implementing the process of Mars Sample Return (the current Perseverance rover mission, *viz.* Farley *et al.,* 2020).

**FIG. 1.**
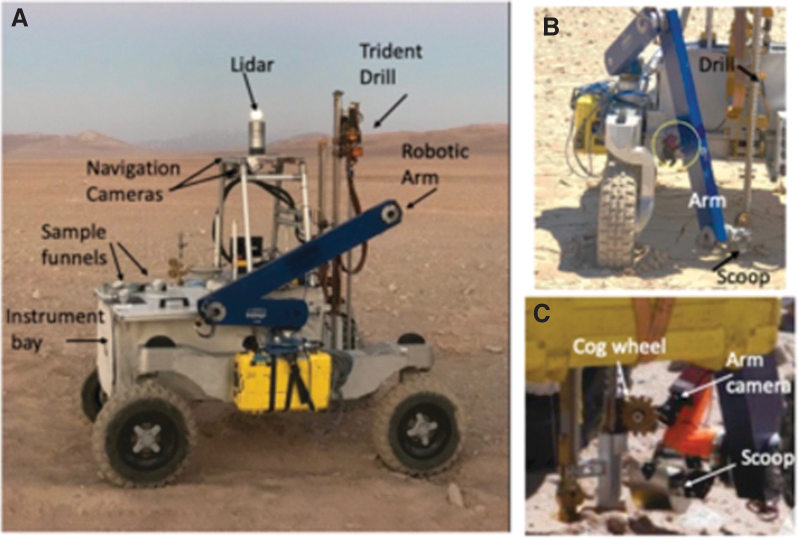
(**a**) ARADS astrobiology exploration system (rover, drill, robot arm, instruments) at the September 2019 tests in Chile. (**b**) Robot arm with scoop extended. (**c**) Cog scraper pushing drilled cuttings from the auger drillstring into the scoop, with air gaps to minimize cross-contamination (Stoker *et al.,* 2023).

Meanwhile, the concept of an astrobiology field laboratory rover needed further development and maturation; hence the Atacama Rover Astrobiology Drilling Studies (ARADS) project concept was formed, inspired in part by the previous AFL concepts. The 2014 concept for ARADS included a medium-sized rover with habitability instruments, including the Phoenix Wet Chemistry Laboratory (WCL) (Kounaves *et al.,* 2009) and an enhanced version of the ExoMars Mars Organic Molecule Analyser (MOMA) Laser Desorption–Linear Ion Trap Mass Spectrometer (LITMS) (Goesmann *et al.,* 2017; Li *et al.,* 2019). ARADS likewise included two biosignature detection instruments: the Spanish Signs of Life Detector (SOLID) immunoassay instrument (Parro *et al.,* 2011a, 2011b; Moreno-Paz *et al.,* 2018) and the Chemical Laptop-derived (Willis *et al.,* 2015) Planetary In Situ Capillary Electrophoresis System (PISCES), while mitigating Planetary Protection–relevant contamination issues. Figure 1 shows the as-built ARADS rover, with its drill and sample transfer arm.

Laboratory tests for developing science operations, instruments, mission technologies, and field experimental protocols often rely on assumptions and models. Terrestrial “analog missions” are a common means to test and validate the aspects of these models and assumptions that may also be observed at Earth sites. Research at terrestrial analogs supports the development and refining of science methods, operations, and improving the maturity of technologies to achieve a higher mission maturity and flight readiness.

The objectives of the ARADS project were to demonstrate the feasibility of roving with drilling missions to Mars and to demonstrate the potential science return for such a mission in the context of the search for evidence of life. ARADS technology goals targeted analog-site demonstrations of automated drilling and sampling, integrated with mobile biosignature detection technologies that would be candidate methods for instruments on future astrobiology missions. ARADS science operations goals were to develop remote science, contamination mitigation, and communications methods to culminate in an AFL-like science operations simulation with time delays, directed by a remote science team.

The ARADS-related papers in this Special Collection reflect the 5 years of development and results of the project, including analog science studies, instrument evaluations, technology demonstration and operations concept development for the culminating ARADS field season in Chile in September 2019. The onset of the COVID-19 pandemic delayed the post-field analyses and publication of this work, and it is worth also noting the earlier ARADS publications (in other venues) for a more complete view of the accomplishments of the ARADS team.

## 2. ARADS Science and Technology Through Analog Research

A planned series of four Mars analog site demonstrations (in Chile's hyperarid Atacama Desert) over the course of 4 years provided a step-by-step guide to the development of the ARADS platform's science capabilities and systems integration, which led to an integrated remote roving mission simulation in the project's final year (phasing shown in Table 1). The extreme aridity and low surface organic content in the hyperarid soils of the northern Atacama Desert in Chile make it one of the most relevant Mars analog environments on Earth. Geological and soil mineralogical evidence suggest that extreme arid conditions have persisted for at least 10–15 million years (Ericksen, 1983), but the sedimentary record indicates the region has been arid since the late Triassic (Clarke, 2006), making it the oldest continuously arid desert on Earth. The driest parts are located between approximately 22°S to 26°S in a broad central depression. Historically, mean annual rainfall in this central region averages <2 mm (McKay *et al.,* 2003). However, in more recent years the region has experienced a number of large rain events that resulted in the temporary formation of lagoons (Azua-Bustos *et al.,* 2018).

**Table 1.** ARADS Project Phasing

Year	Instruments	Ground truth analysis	Technology
2016	WCL, SOLID	Organics, salts, microbiology	Drill, instruments not integrated; SOLID, WCL baselines as standalones; no rover in field
2017	WCL, PISCES, SOLID	Organics, salts, microbiology	Drill and rover integrated and tested; instruments standalone; baseline for PISCES
2018	WCL, PISCES, SOLID	Organics, salts, microbiology	Drill, rover, sample handling integrated with onboard instruments, manually operated
2019	LITMS, WCL, PISCES, SOLID	Organics, salts, microbiology, biosignature spatial distributions	Drill, rover, sample handling automated; instruments integrated and fed onboard, remote science operations; standalone for LITMS

**FIG. 2.**
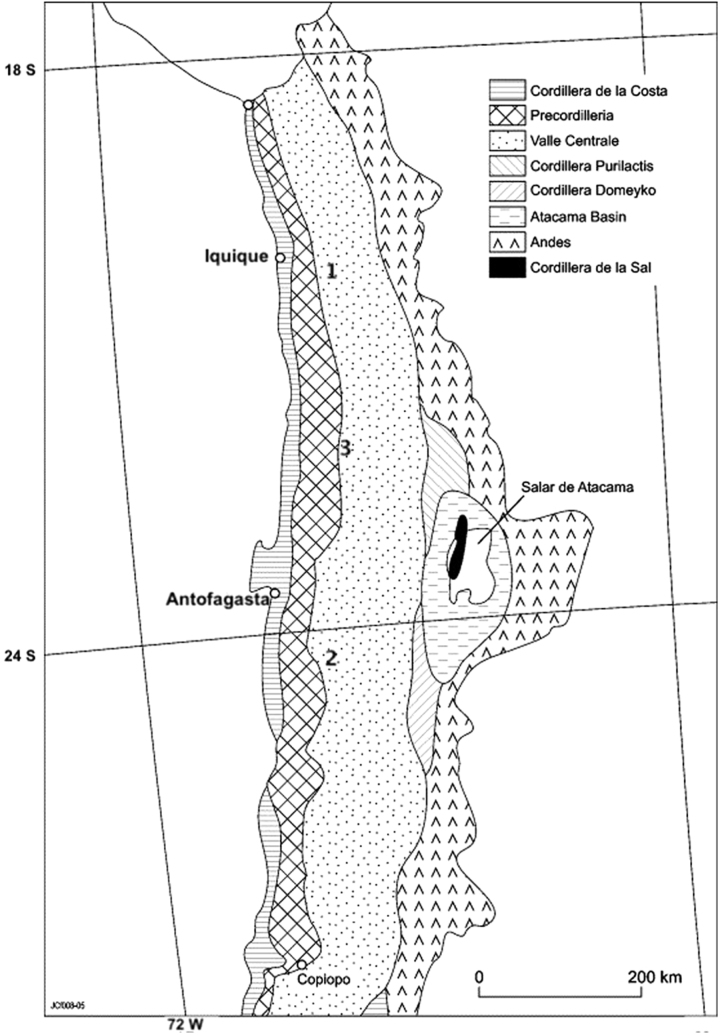
Initial three Atacama Desert sites considered and surveyed for ARADS tests (map annotated from Clarke [2006]). 1 = Salar Grande, 2 = Yungay, 3 = Maria Elena South.

Influencing the choices of Chilean test areas were analog field studies in the Atacama prior to ARADS, including past deployments and traverses by the robotic “Nomad” rover (Cabrol *et al.,* 2001) and the drill-bearing Life In The Atacama (LITA) rovers (Warren-Rhodes *et al.,* 2019). Prior biosignature and microbial extremophile studies by ARADS science team members and their past field experience would support ground-truthing and metrics at several Atacama sites. Of the three initial Atacama Desert areas initially considered for ARADS and surveyed in Year 1 (see Fig. 2), the diversity of hyperarid environments and existing support infrastructure (an otherwise-disused research station owned by the University of Antofagasta) in the Estacion Yungay region (Site 2) led to its selection for the ARADS engineering and instrument field demonstrations and as a base of operations in Years 2–4.

**FIG. 3.**
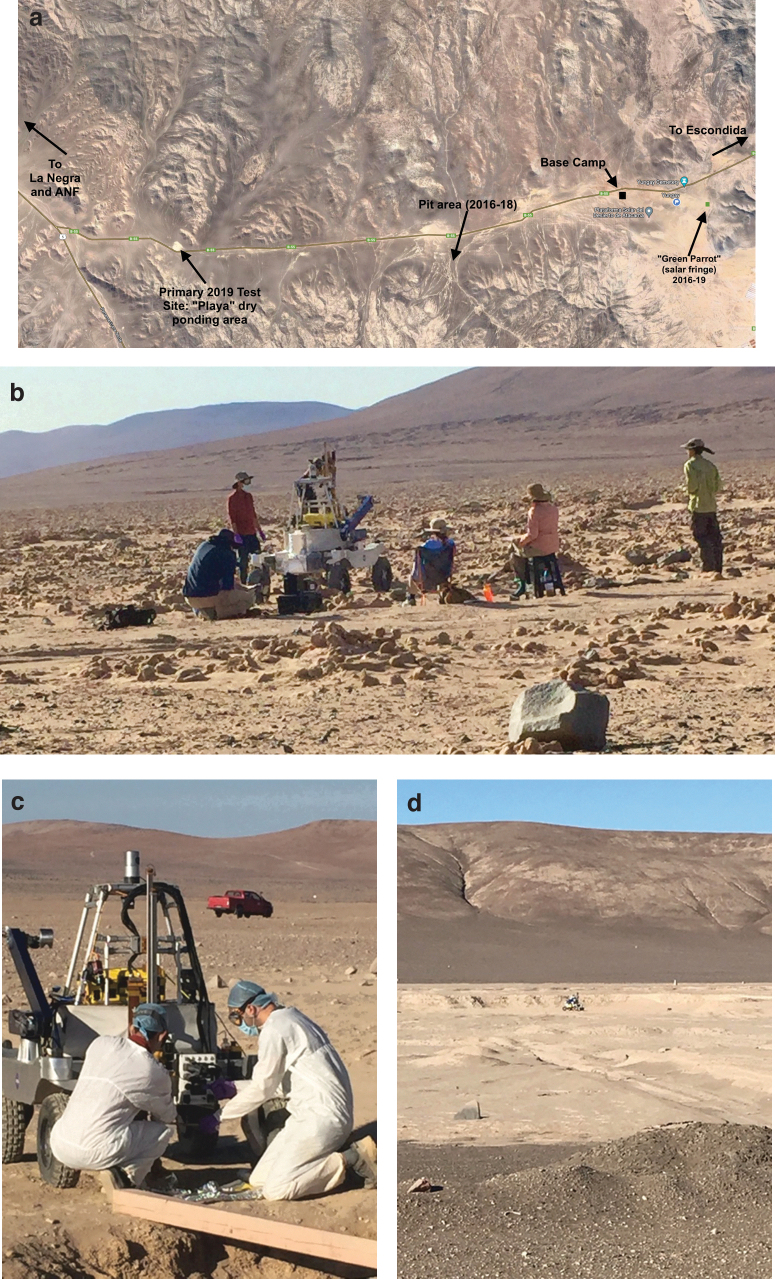
(**a**) Site 2 overhead context, showing the Yungay base and the test sites. (**b**) The Green Parrot site, at the edge of a salar only 2 km from the Yungay base camp, supported integration and testing of the drill, sample transfer, and instruments. This 2017 photo shows initial SOLID onboard testing together with the drill and arm. Halite nodules are apparent on the surface nearby. (**c**). In 2016–18 the drilled samples acquired adjacent to a human-excavated pit provided collaborating data for ongoing studies there. In this photo, the rover and drill are undergoing a multistep cleaning protocol prior to sampling. (**d**). In September 2019 the ARADS system autonomously navigated to target locations at the Playa kilometer-wide site for sampling and analysis, as directed by twice-daily command sets sent remotely from the science team.

Figure 3a shows the geographical context of the ARADS field studies in the Estacion Yungay region, centered at roughly 24°S, 70°W about 80 km southeast of the city of Antofagasta in Chile. The area shown in Fig. 3a as “Green Parrot” (Fig. 3b) lies about 1.5 km east of the University of Antofagasta research station, on the opposite (eastern) edge of a salar, and had previously been studied by Co-Investigator DiRuggiero, her colleagues, and students (Robinson *et al.,* 2015). This prior ground-truth data, at a location conveniently near the ARADS base of operations, led to Green Parrot's use as a checkout and shakedown site for the ARADS drills, the “KREX2” research rover (see Heldmann *et al.,* 2016), and instruments in the 2016–19 deployments, including the March 2019 standalone field test of the LITMS laser spectrometer. Approximately 8 m west of the basecamp was an area of consolidated sediment and halite layers, an area with an existing manually dug 2 m deep pit from prior studies (Finstad *et al.,* 2014). The existence of independent stratigraphic biosignature profiles, to 2 m depth, supported the testing of the ARADS drill hardware, software, and system integration with the rover and instruments. The “Pit” area (Fig. 3c) was the primary field science study area in Years 1–3 (2016–18), and ARADS sample data obtained from drilling depths to 2 m was used together with parallel manual pit sample data in the PhD dissertation by Wilhelm (2017).

While developing the ARADS system in previously studied Atacama sites provided a useful control and yardstick for evaluating the results obtained, for the final-year demonstration (2019) a novel site was chosen, with the ground truth and field studies conducted in parallel. Without *a priori* site knowledge, the ARADS system could then demonstrate its capability for exploration and detection of novel results, correlated later with the separate-but-collocated manual field study results as a control. The “Playa” (Fig. 3d) is a dry, episodically ponded area approximately 1 km in diameter, roughly 20 km west of the base camp. Parallel manual field studies provided a control. More detail regarding traverses and field operations at the Playa site are discussed by Stoker *et al.* (2023) in this issue.

## 3. Proposed Goals and Objectives

The astrobiological scientific objectives motivating ARADS were to understand the mobility and distribution of soluble salts, organic compounds, biosignatures, and extant life in the extremely dry soils in the Atacama, as an analog system to Mars, down to the 2 m depths proposed for exploration with the ExoMars rover (or Icebreaker). ARADS proposed a system capable of addressing three hypotheses, which drove its instrumentation and system design:

**H1**. The distribution of organic compounds in hyperarid soils would be heterogeneous near the surface and controlled by photochemical and biological processes.
**H2**. Organics compounds in hyperarid soils would accumulate with depth due to transport and protection from photochemistry.
**H3**. Increasing values of organic compounds and soluble salts with depth would be correlated with increasing concentrations of cells and a shift in the composition of the microbial communities.

Based on these science goals and hypotheses, the technical and operational objectives of the proposed research were to

(1) Develop an engineering system that could traverse rugged Mars-like terrain and carry a drilling system,
(2) Remotely operate this integrated mobile system to select and deploy the drilling system,
(3) Drill and acquire relevant samples from discrete depths for sample analysis,
(4) Analyze the samples with flight prototype instruments, and
(5) Demonstrate science team interaction in the mission through a remote interface.

## 4. ARADS Development Progression

Our ARADS field campaigns then began with baseline field science and individual component and instrument tests in Year 1, with increasing levels of integration and automation leading to fully autonomous rover/drill site survey, selection and drilling, and sample analysis operations by Year 4. Table 1 summarizes the activities by year.

The iterative, incremental build-and-test approach evident in Table 1 led to several design changes. The originally proposed sampling drill was the Honeybee Robotics “Icebreaker-3” rotary-percussive drill, an upgraded LITA drill. Separate analog field tests of this drill in 2015 in Río Tinto, Spain (Glass *et al.,* 2016; Sánchez-García *et al.,* 2020) found that this drill lacked sufficient applied torque and percussive energy to make headway in hard rocks or consolidated sediments. Working with the VIPER program (then called Resource Prospector) and Honeybee, joint requirements and ARADS sampling issues drove the 2015–16 design of a new drill, “The Regolith and Ice Drill for Exploring New Terrain” (TRIDENT) with greater power and penetrative ability. The ARADS drill (Fig. 1) was the very first TRIDENT delivered to NASA (in November 2016) and was effectively beta-tested by ARADS at NASA Ames Research Center (December 2016) and in Chile (February 2017), leading to small TRIDENT design changes in its cabling and avionics to improve its reliability.

**FIG. 4.**
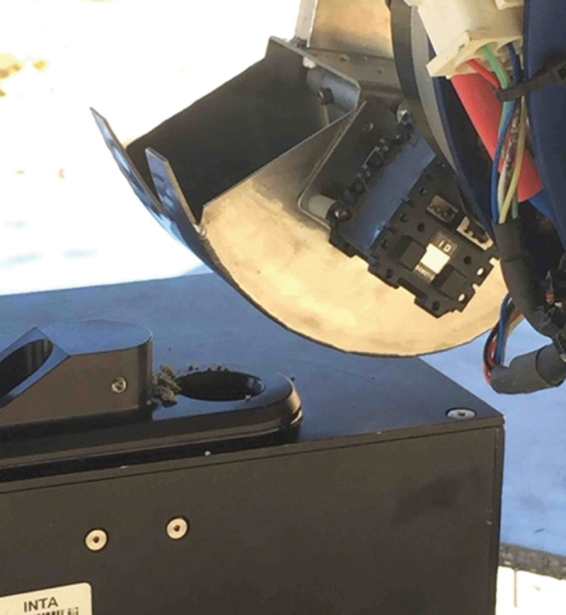
Sample from the ARADS scoop, in a test delivery to the SOLID SPU.

On the ARADS platform (Fig. 1), drilled sample was to be transported from the drill to instrument intakes by a robot arm with a metering-capable bucket or scoop as an end-effector. An existing robot arm and several scoop designs were tested in the first field season (March 2016) but lacked sufficient pointing accuracy for reliable sample deliveries. Subsequently, a new arm, derived from the Maxar arm from the Phoenix mission, was designed and delivered to ARADS by Maxar. A new scoop, scaled down from the Phoenix Icy Soil Acquisition Device design (Bonitz *et al.,* 2008), but with a progressive internal paddle design providing 5gm-level sample metering capability, was built at NASA Ames Research Center (Fig. 4) and integrated with the Maxar arm and rover avionics. The arm and scoop were tested and (with the addition of a wrist camera) met requirements after the 2017 field season (Dave *et al.,* 2018).

Instrument maturation and progression went through three steps: (a) initial standalone testing and operation in Chile, with performance confirmed with comparison to prior results and/or post-season laboratory evaluation of field samples; (b) integrated instrument operation demonstrated at NASA Ames with the KREX2 rover and its avionics and control software; (c) demonstration of onboard instrument operation in Chile.

Development and deployment of the Laser Desorption–Linear Ion Trap Mass Spectrometer (LITMS) prototype was connected to the parent MOMA instrument development for the ExoMars mission, and the LITMS team unavoidably experienced ExoMars-related schedule delays. These delays ultimately led to the Yungay field demonstration of the LITMS being performed independently of the rest of ARADS, as a standalone field test in March 2019.

## 5. ARADS System Components

### 5.1. Honeybee Robotics TRIDENT 1 m drill

Building on two decades of midsize rover development as well as several generations of automated drill design between NASA Ames and industry partners, the ARADS project combined the NASA KREX2 300 kg rover prototype with a 1 m rotary-percussive drill from Honeybee Robotics—a drill design jointly developed by ARADS with the then Resource Prospector project. This TRIDENT drill design (Zacny *et al.,* 2017, 2021) is also the baseline design for the VIPER lunar prospecting mission (planned for launch in late 2024) and the Polar Resources Ice Mining Experiment-1 (PRIME-1) lunar drilling experiment (expected to fly on an Intuitive Machines lander in mid-2023).

### 5.2. Mobility platform: KREX2 rover

The Ames KREX2 rover (Fig. 1) is a 300 kg class midsized 4-wheeled prototype planetary surface rover, one of the line of NASA Ames Research Center's “K” series of rovers, and was first deployed for the Mojave Volatiles Prospector project (Heldmann *et al.,* 2016). For ARADS, the KREX2 research rover provided mobility and autonomous navigation services to a <100 kg instrument payload, powered the instruments and subsystems, and provided a local communications link used in ground support of the instruments.

During the September 2019 ARADS field deployment, Honeybee also installed on KREX2 and evaluated its Apollo-style “Stinger” geotechnical sensing penetrometer (Mank *et al.,* 2021), in parallel with the existing TRIDENT drill. Another ARADS-opportunistic rover test assessed the performance of a novel rover wheel, a tensegrital design from Protoinnovations (Crowther *et al.,* 2022). This surface mobility wheel design applied tensegrity principles (geometric and mechanical) to achieve the equivalent load-distributing and compliance properties of a pneumatic tire while using only space-qualified materials.

### 5.3. Sample transfer arm/scoop

A Maxar-developed robot arm (derived from past Mars InSight and Phoenix arms, and visible in Fig. 1) with a Phoenix-derived scoop transported cuttings from the drill to the instrument payload deck on the KREX2 rover, as shown in Fig. 1. The ARADS project instruments are carried opposite the drill and arm, mounted on the KREX2 rover payload deck. The process of drilling, bringing up sample, catchment in the scoop, transport of the sample over to the instrument inlets, and actuated sample transfer into the inlets was fully automated and monitored by onboard software.

### 5.4. Onboard autonomy software

The ARADS onboard autonomy software (Glass *et al.,* 2018; Stucky *et al.,* 2018) performs fault monitoring, safing, sequencing, and real-time tasking and scheduling, using the NASA developed Plan Execution Interchange Language (PLEXIL). Drill and sample handling systems must be able to perform completely hands off and operate for hours at a time, between periodic uplinks and downlinks. The ARADS architecture (Stucky *et al.,* 2018) has a central software executive with a diagnostic software module, which work together while monitoring and adapting to changing rover, drill, and instrument conditions and states. Kinematic and dynamic models in the onboard software provide for detection and adjustment of the drilling and sampling angles to accommodate the orientation of the rover wheels and chassis.

### 5.5. Signs of Life Detector (SOLID)

**FIG. 5.**
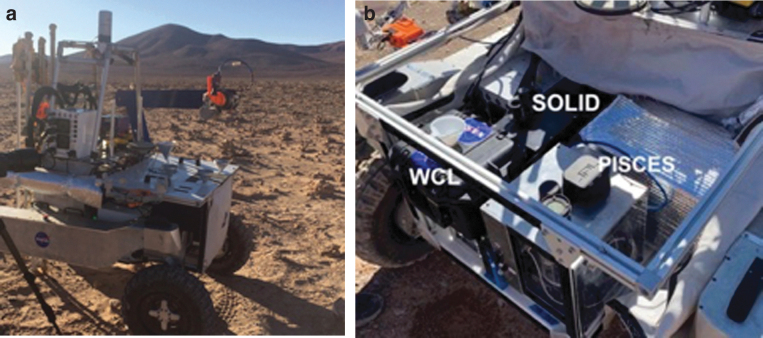
(**a**) Since the drill was mounted on the aft side of the rover (left), the robot arm and scoop transported the drilled samples laterally to the inlet funnels (shown right). (**b**) ARADS instruments (SOLID, PISCES, and WCL) as installed September 2019 in the rover forward payload deck.

The prototype SOLID instrument (from the Instituto Nacional de Técnica Aeroespacial's [INTA] Centro de Astrobiología [CAB] in Spain) tested on ARADS and on a sister Life-detection Mars Analog Project (LMAP) lander (Sánchez-García *et al.,* 2020) is divided into two main units: the sample preparation unit (SPU), which uses ultrasonication to extract biomolecules from sample into a buffer solution and is connected to the sample analysis unit (SAU) that bears the antibody microarray biosensor (the Life Detector Chip or LDChip) for immunological assays (Moreno-Paz *et al.,* 2023). Figure 5 shows both parts of SOLID installed on the rover payload deck. SOLID is based on antibody microarray immunoassays and can detect a broad range of molecular size compounds, from amino acids to whole cells quickly (from minutes to 3 hours, depending on the type of immunoassay; see Parro *et al.* [2011a, 2011b] and Moreno-Paz *et al.* [2018]). The SPU can accept either solid (soil, ground rock, or ice) or liquid samples.

### 5.6. Planetary In Situ Capillary Electrophoresis System (PISCES)

The Planetary In Situ Capillary Electrophoresis System (PISCES) instrument (from NASA's Jet Propulsion Laboratory) is an end-to-end chemical analyzer that is capable of ingesting soil samples, obtaining extracts, producing chemical inventories of amino acids present at parts-per-billion levels and determining their chiral ratios. Onboard the ARADS rover, PISCES comprised a microchip electrophoresis analyzer (Mora *et al.,* 2020) coupled to a subcritical water extractor (Kehl *et al.,* 2019). This capability is valuable for life-detection missions to habitable environments on Mars and icy worlds. PISCES contains a front-end extraction apparatus that accepts soil samples and produces liquid extract materials, as described by Kehl *et al.* (2019). This fluid output is first analyzed for pH, conductivity, and ionic content using a customized microfluidic flow injection analyzer system (Mora *et al.,* 2020) visible in Fig. 5. Liquid extracts are then passed to a microchip electrophoresis analyzer (known as the Chemical Laptop), where the chiral amino acid analysis is performed as described by Willis *et al.* (2015). Field results as delineated by Mora *et al.* (2020) showed five amino acids detected in field operations in three separate drilled samples, with a fourth sample inconclusive.

### 5.7. Wet Chemistry Laboratory (WCL) Testbed

The Wet Chemistry Laboratory (WCL) Testbed (NASA Ames) is a version of the Jet Propulsion Laboratory (JPL) instrument that was used during the Mars Phoenix Mission to characterize landing site habitability by measuring aqueous soluble components in martian surface material. The WCL testbed is equivalent to the WCL flight instrument (Kounaves *et al.,* 2009) except for the electronics, which although functionally equivalent are not packaged for flight. WCL was deployed in the Atacama Desert as part of several ARADS campaigns to examine soil chemistry in the content of habitability and biosignature preservation. The funnel used to feed the testbed is visible in Fig. 5, installed on the rover.

### 5.8. Linear Ion Trap Mass Spectrometer (LITMS)—laser desorption

The ARADS Linear Ion Trap Mass Spectrometer (LITMS), from NASA Goddard Space Flight Center, interrogates the chemical composition of geological samples acquired from Atacama surface materials. Central to the instrument is a miniaturized linear ion trap mass spectrometer, whose design is similar to that of the MOMA flight instrument on the ExoMars rover mission. During LITMS operations, as described by Brinckerhoff *et al.* (2017) and Li *et al.* (2019), the sample is maintained at Mars ambient pressures (*i.e.,* 4–8 torr, primarily CO_2_) and irradiated by a solid-state, Nd:YAG laser source generating UV light (266 nm wavelength) in 1 ns pulses at a repetition rate up to 100 Hz, and with adjustable output energy (250 μJ maximum). The LITMS instrument, designed to operate in a Mars environment, required samples to be manually placed inside its own miniature Mars environmental chamber (“Mars-Box”), secured, then pumped-down to Mars-ambient and backfilled with a model Mars gas mixture. The Mars-Box onboard would have been accommodated adjacent to the KREX2 payload deck, adequate for manual sample access and for the required vacuum line and gas feeds.

### 5.9. Cleaning protocols (initial and final)

Standalone instrument field evaluations began in the first ARADS field deployment in February 2016, and the field science requirements for uncontaminated drilled samples led to the design of a multistage deep chemical cleaning protocol. Bulk debris cleaning was routinely followed by applications of ultrapurified water, methanol, acetone, and hexane to surfaces that come into direct contact with samples, such as the drill string, arm scoop, or instrument inlets. Field heat sterilization was considered but not used as heat sterilization would not remove organic contaminants. Figure 3c illustrates the multistage protocol underway prior to drilling and sampling, shown at the Pit study site.

Given the sensitivities of both a simulation running on prototypes and brassboard equipment (requiring out-of-simulation replenishment, servicing, and maintenance by field staff) and looking for local biosignatures and extant Atacama organisms (while minimizing and controlling external cross-contamination), a mission simulation contamination control plan was developed in early 2019 and was tested/calibrated in dry-runs beforehand. Details are discussed in the work of Bonaccorsi *et al.* (2023). The *in-situ* real-time adenosine triphosphate (ATP) luminometric assay (invisible to the simulation) was used to monitor the efficiency of the cleaning/microbial reduction protocols and for bioburden monitoring of the ARADS hardware during the simulation. Beginning- and end-of-day ATP bioluminescence assays at rover stations also provided an indirect indicator of the total biomass latent in the unearthed drilled sample, as well as from windborne dust deposits and their potential source areas. More contamination assessment and management details are given in the work of Bonaccorsi *et al.* (2023).

## 6. Communications and Tasking Design

**FIG. 6.**
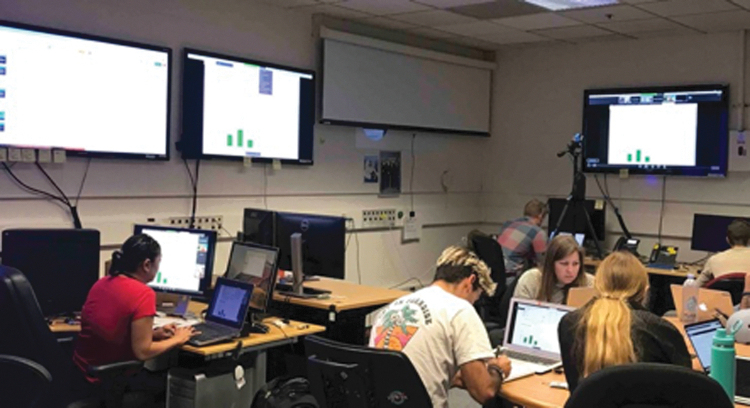
Remote Mission Operations Center (at NASA Ames Research Center, supported from Spain, GSFC, and JPL), shown in use during September 2019 ARADS operations in Chile.

**FIG. 7.**
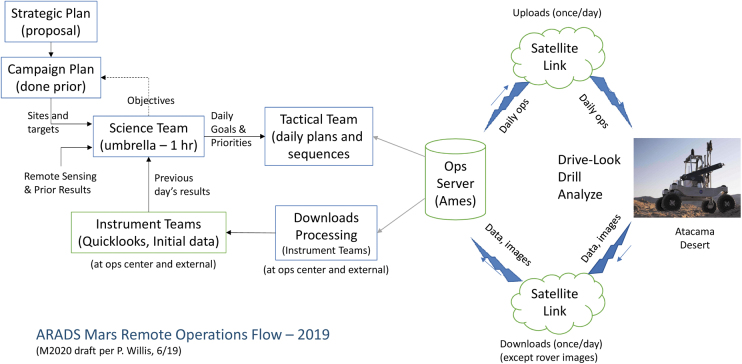
Remote Mission Operations flow, derived from M2020 concepts (Glass *et al.*, 2021).

The ARADS project concluded its final field season in September 2019 with a 6-day remote operations simulated mission, at a new field location (not studied in previous rover-based drilling and sampling) to leave open the possibility of novel observations. The PISCES instrument lead and co-investigator (P. Willis, also a Mars 2020 science team member) provided insight into the evolution of the daily operations flow planned for the Mars 2020 rover mission operations (Farley *et al.,* 2020). The ARADS operations flow was adapted to an analog-field-scale operation with a remote Mission Operations Center (Fig. 6) at NASA Ames; this flow included uploading daily commands and sequences to the ARADS platform in Chile via satellite communications, receiving daily data downloads in return. The returned data were the basis of instrument quick-looks guiding generation of tactical plans in the following day's Science Team meeting at NASA. Figure 7 shows the flow of strategic and tactical planning.

Current low-cost data tools (Slack, Google Docs) were used for establishing easily configurable, low-latency data channels and repositories for raw and processed datasets, sample and site information, and quick-look material.

Remote operations planning also required definition of roles and responsibilities for remote and field teams before and during the simulated ARADS mission operated remotely from NASA Ames. The science team members were selected based on knowledge of relevant biology and geology, but limited experience exploring the candidate analog field site(s). Roles were articulated for a flight lead, science team lead, and tactical team leads for providing operations support. Remote sensing data and platform-scale (navigation and engineering) sensing helped determine the drilling and thence sampling targets.

In the field, autonomous rover, drilling, and sample-transfer operations were human-supervised by a field engineering team, wearing protective equipment, who also cleaned and decontaminated the sample-contact chain and (at a distance) implemented operating safety with emergency power cutoffs if necessary. These “black box” functions were out of simulation and invisible to the remote science team at NASA Ames, likewise the after-hours daily field maintenance functions (recharging rover batteries, replenishing instrument consumables, cleaning/microbial reduction and verification, minor instrument repairs, removal and curation of samples for later studies). Engineering data from the rover (including *in situ* instrument engineering data) was monitored from a field test support facility set up a few hundred meters away.

## 7. Daily Operations Flow—2019 Demonstration

**FIG. 8.**
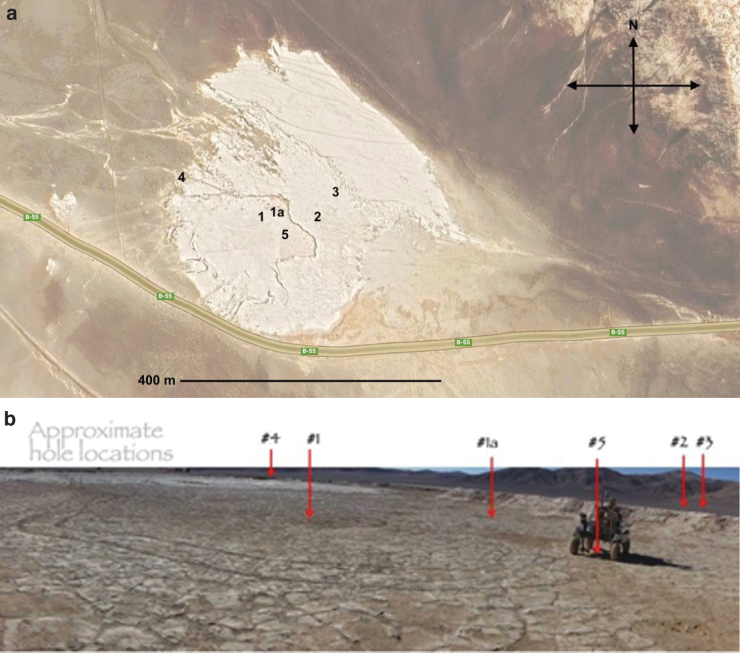
(**a**) the “Playa” test site (24°06'08.3″S, 70°08'17.5″W) about 22 km east from the base of field support at Estacion Yungay. (**b**) North view of the dry basin, with approximate 2019 borehole locations indicated.

A goal of the final 2019 demonstration of the ARADS project was to use its instrumented rover to investigate, actually exploring a novel location where the provenances and subsurface layering would be *a priori* unknown (as would likely be true of a planetary mission). Some weeks prior to field arrival, the science team examined the available remote sensing data (typically Google Earth with 60 cm resolution, roughly comparable to HiRISE at Mars) and any published data regarding their selected “landing” zone candidate. Basic operation modules were agreed upon, wherein each module constituted a set of commands and a Mars operations day timeframe. Automated planning software then arranged these operations into a strategic plan and then a campaign plan to prioritize the rover's movements after arrival. Target points of interest were prioritized with possible traverse paths linking them, avoiding one blackout zone where a parallel manual field study (K. Warren-Rhodes 2022, unpublished data) was in progress, and allowing a window for daily command uplinks. Figure 8 shows the initial campaign area with the selected sampling locations indicated. More details of the remote operations are given by Glass *et al.* (2021) and Stoker *et al.* (2023).

During the campaign, the science team (meeting at NASA Ames, and supported virtually from JPL and from the Instituto Nacional de Técnica Aeroespacial's [INTA] Centro de Astrobiología [CAB] in Spain) considered the prior day's results (quick-look reports, QLs) in an initial morning meeting and would decide whether to proceed along the campaign plan, stay at the current location, or pursue another potential science opportunity nearby. A “tactical team” would then formulate the day's series of command modules to be relayed to the field in Chile. In the field (a 4 h time difference), the rover would meanwhile be removed from overnight storage, prepared, and cleaned, with instruments (re-)installed as necessary. Once the day's command sequences were received in the field, the day's deployment, drilling, and analyses would commence. Meanwhile, the science team (in Spain and California) would have adjourned for the day. Toward the end of the field-timezone day, initial *in situ* results were uplinked to a NASA Ames–based file server and were compiled into QLs for each instrument (including the drill) for the following day's science team meeting. An exception was the PISCES instrument, whose sensitivity to external vibrations required it to finish its sample processing early the next field day (5 a.m. local) with no other rover activity in parallel—but still in time for the PISCES team to produce its QL before the science team meeting convened.

Our metrics for evaluating the ARADS simulation included the following: (a) Instrument cycle times (durations); (b) Whether usable results were produced by a given instrument; (c) If operator interventions (out of sim) were necessary; (d) Number of remote faults and recoveries; (e) Rover, drill, and transfer arm/scoop action durations, time necessary to execute; (f) Drilling depth, and holes drilled per target site; and (g) Daily bioburden levels (ATP surface swab assay tracking values). Together these durations showed that deep drilling and the associated sample handling constitute about 25% of operations, with the bulk of rover time on-station used during stationary sample analysis by the instruments during a given simulated Mars day.

## 8. ARADS-Supported Astrobiology Field Studies

Over the duration of the ARADS project, astrobiology-directed field science was conducted at the Atacama sites. This work was novel and publishable as well as providing a valuable local control and ground truth for correlation with the data and results from the ARADS instruments. The Atacama biosignatures (discussed previously in *Astrobiology* by ARADS co-investigator Wilhelm *et al.* [2018]) followed Wilhelm's PhD dissertation (Wilhelm, 2017) that studied a subset of biosignatures, namely lipids and amino acids, found geographically and at depth in the Atacama as markers for microbial adaptation and growth. Wilhelm's Yungay-area samples (Pit area) also served as ground-truth controls and drove the initial field shakeout objectives for the initial TRIDENT drill evaluation in 2017. Lipid biosignatures were found to increase with depth generally, supporting ARADS **H2** (accumulation with depth, away from ionizing surface radiation). While most of Co-Investigator DiRuggiero's team's research was focused on microbial communities at Site 1 (Salar Grande), an unusual rainfall event in the Site 2 (Yungay) area prior to the 2017 ARADS deployment provided a rare opportunity, discussed in peer-reviewed papers led by her graduate student Uritskiy (2019, 2020a, 2020b), to study the microbial community compositional shifts before and a shift after the deluge, and then a slow population compositional recovery (in subsequent years).

The unanticipated availability of field-capable nanopore instruments such as the MinION in 2016–17 led to an ARADS-sponsored field evaluation to determine the nanopore instrument detection limit of DNA and RNA contained in samples from the Site 1 test area (Green Parrot), as described by Bywaters *et al.* (2016). DNA field-sequenced from drilled endolithic halite samples successfully identified cyanobacteria and associated heterotrophic bacteria. Experience from this initial field test influenced the subsequent interest in, and development of, solid-state nanopore technology (Bywaters *et al.,* 2019), which is less thermally constrained.

## 9. External Impacts

Even as the final field season concluded in Chile in late 2019, the appearance of COVID-19 in early 2020 and the subsequent stay-at-home orders at NASA and universities during the pandemic caused significant delays in completion of sample analysis, ground-truth sample processing, instrument data processing, and assessment of the ARADS system performance and operations. The papers in this ARADS Special Collection were significantly delayed in turn. Results from two of the four ARADS instruments are consequently not included in this collection: the PISCES team managed to finish their manuscripts and submitted them (Kehl *et al.,* 2019; Mora *et al.,* 2020) prior to JPL's pandemic shutdown in 2020. But the WCL brassboard instrument field data and results are unlikely to be published at all (as the term of the postdoctoral associate responsible for the instrument and WCL field data analysis ended during the pandemic suspension).

## 10. Compiled ARADS Special Collection

The papers in this ARADS Special Collection reflect field studies, operations, and technology that were fielded in the Atacama Desert in four successive years, culminating in the 2019 deployments. The papers in the Special Collection are a presentation of the ARADS project's results.

These results include two of the four ARADS onboard instruments. The SOLID instrument and its offline Life Detector Chip (LDChip) were participants in each of the four ARADS seasons, with initial rover and drill/arm integration as early as the 2017 season. As indicated in the work of Moreno-Paz *et al.* (2023), the SOLID-LDChip immunoassay instrument assembly remote unequivocally detected microbial biosignatures associated with known species, in a changing pattern with depth (addressing the original ARADS **H3** hypothesis). The LDChip antibody microarray results in this Special Collection paper are complemented with a comprehensive post-season laboratory analysis of the curated drilled field samples, including metaproteomics and DNA sequencing, as well as XRD, mass spectroscopy, and ion chromatography, which support ARADS **H1** (near-surface heterogeneous distribution of organic compounds).

The intended onboard mass spectrometer for ARADS, the Linear Ion Trap Mass Spectrometer (LITMS), is based on the ExoMars Mars Organic Molecule Analyser (MOMA) instrument (Li *et al.,* 2018). Rescheduling of the ExoMars launch date conflicted at times with LITMS ruggedization efforts and meant that the engineering-unit prototype MOMA avionics were no longer available in time for ARADS rover installation. Despite these challenges, Castillo *et al.* (2023) describe their modifications that enabled the brassboard (rack-based) version of the LITMS to be brought to Chile and field tested at Green Parrot in March 2019 with drilled and transferred sample. While the LITMS instrument did not participate in the September 2019 tests, this ARADS Special Collection paper also provides post-season results from LITMS spectroscopic analyses of curated drilled samples from the September deployment.

The combined operation of the ARADS instruments—drill and rover, remotely commanded in a simulated mission—demonstrated the efficacy of this surface exploration prototype. Stoker *et al.* (2023) chronicle the September 2019 Year 4 ARADS field deployment, reporting on the elements of the remote operations demonstration, field tests with the integrated drilling rover, and initial instrument results. A California-based science team sent instructions daily to a Chile-based field team that remotely supported and observed the drill, sample delivery arm, and onboard instruments. Stoker describes the selection process for the “Playa” field site by the remote science team and the complexity of setting and transmitting daily drill targets within Mars-like communication constraints. A complementary discussion of metrics and performance during the September 2019 ARADS remote operations simulation (Glass *et al.,* 2021) addresses how commands and data were packaged and transferred by twice/daily (Deep Space Network–like timing) uplink and downlink from the NASA Ames control center. Operational results from these tests suggest that science operational efficiency improved with experience, in roving, contamination control, navigation/targeting, drilling, and sample transfer operations during the course of the weeklong simulation.

A critical aspect of future missions in search of biosignatures beyond Earth is the tracking and maintaining sample-contact cleanliness to address both concerns of planetary protection: forward contamination and minimizing the possibility of false-positive detections from exogenous contaminants introduced into the sample analysis (Kminek *et al.,* 2019). Bonaccorsi *et al.* (2023) present the field Contamination Control Strategy and Implementation (CCSI) developed for the Sample Handling and Transfer System (SHTS) hardware and practices used in the 2019 ARADS tests. Bonaccorsi *et al.* (2023) also describe the primary contamination issues experienced during bioburden monitoring (*e.g.,* false negatives incurred during cleaning verification of drill hardware, hardware geometry, and surface roughness), the mitigation practices developed (prevention, cleaning/microbial reduction and assessment by co-analysis with LDChip immunoassay), and their applicability under Atacama field conditions.

Furthermore, the distribution of ATP biomarker representative of metabolically active microbial biomass in air samples and Playa sediments is evaluated as a potential anthropogenic and environmental contaminant to the collecting hardware and drilled samples. Finally, varying levels of external anthropogenic activity are compared as an example to the biosignature detection results from one of the three ARADS onboard instruments (SOLID). The subset of the results taken from the “Playa” site (manually sampled and analyzed in post-season laboratory instruments) also served as an independent parallel “ground truth” control on the September 2019 data from the ARADS rover instruments.

## 11. Summary

The search for signs of past or extant life and the fostering of human exploration on Mars will require a mobile survey capability to detect biosignatures (if any exist) as well as potential resources. The ARADS team sought to conduct astrobiologically relevant investigations with candidate flight instruments in the hyperarid Mars analog environment of the Atacama Desert. During operations, both onboard automation and human-machine interactions were noted and improved. The papers in this Special Collection, together with the other referenced ARADS-related publications, provide a view of the issues and benefits of field testing these methods in astrobiologically relevant environments prior to proposing them for spaceflight. *Astrobiology* was, therefore, a logical choice to host the ARADS Special Collection.

With Mars Sample Return underway, the most recent Decadal Survey (NAP, 2022) called for attention next to the potentials of Mars astrobiology—and called for an initial Mars Life Explorer mission prior to the arrival of human explorers. With advances in commercial space launch capabilities and reduced costs to orbit, humans may arrive on Mars within a decade, so “learning how to survey for life” becomes a critical-path Mars precursor in the not-so-far future.

## Abbreviations Used

AFL: Astrobiology Field Laboratory
ARADS: Atacama Rover Astrobiology Drilling Studies
ATP: adenosine triphosphate
JPL: Jet Propulsion Laboratory
LDChip: Life Detector Chip
LITA: Life In The Atacama
LITMS: Linear Ion Trap Mass Spectrometer
MOMA: Mars Organic Molecule Analyser
PISCES: Planetary In Situ Capillary Electrophoresis System
QLs: quick-look reports
SOLID: Signs of Life Detector
SPU: sample preparation unit
TRIDENT: The Regolith and Ice Drill for Exploring New Terrain
VIPER: Lunar Volatiles Investigating Polar Exploration Rover
WCL: Wet Chemistry Laboratory
